# Evaluation of Telemedicine Use for Anesthesiology Pain Division: Retrospective, Observational Case Series Study

**DOI:** 10.2196/33926

**Published:** 2022-04-27

**Authors:** Laleh Jalilian, Irene Wu, Jakun Ing, Xuezhi Dong, Joshua Sadik, George Pan, Heather Hitson, Erin Thomas, Tristan Grogan, Michael Simkovic, Nirav Kamdar

**Affiliations:** 1 Department of Anesthesiology and Perioperative Medicine University of California, Los Angeles Los Angeles, CA United States; 2 Department of Anesthesiology, Critical Care, and Pain Management Hospital for Special Surgery New York, NY United States; 3 University of California Los Angeles Telehealth University of California Los Angeles Health Los Angeles, CA United States; 4 Gould School of Law University of Southern California Los Angeles, CA United States

**Keywords:** COVID-19, pain management, telemedicine, cost savings, patient satisfaction

## Abstract

**Background:**

An increasing number of patients require outpatient and interventional pain management. To help meet the rising demand for anesthesia pain subspecialty care in rural and metropolitan areas, health care providers have used telemedicine for pain management of both interventional patients and those with chronic pain.

**Objective:**

In this study, we aimed to describe the implementation of a telemedicine program for pain management in an academic pain division in a large metropolitan area. We also aimed to estimate patient cost savings from telemedicine, before and after the California COVID-19 “Safer at Home” directive, and to estimate patient satisfaction with telemedicine for pain management care.

**Methods:**

This was a retrospective, observational case series study of telemedicine use in a pain division at an urban academic medical center. From August 2019 to June 2020, we evaluated 1398 patients and conducted 2948 video visits for remote pain management care. We used the publicly available Internal Revenue Service’s Statistics of Income data to estimate hourly earnings by zip code in order to estimate patient cost savings. We estimated median travel time and travel distance with Google Maps’ Distance Matrix application programming interface, direct cost of travel with median value for regular fuel cost in California, and time-based opportunity savings from estimated hourly earnings and round-trip time. We reported patient satisfaction scores derived from a postvisit satisfaction survey containing questions with responses on a 5-point Likert scale.

**Results:**

Patients who attended telemedicine visits avoided an estimated median round-trip driving distance of 26 miles and a median travel time of 69 minutes during afternoon traffic conditions. Within the sample, their median hourly earnings were US $28 (IQR US $21-$39) per hour. Patients saved a median of US $22 on gas and parking and a median total of US $52 (IQR US $36-$75) per telemedicine visit based on estimated hourly earnings and travel time. Patients who were evaluated serially with telemedicine for medication management saved a median of US $156 over a median of 3 visits. A total of 91.4% (286/313) of patients surveyed were satisfied with their telemedicine experience.

**Conclusions:**

Telemedicine use for pain management reduced travel distance, travel time, and travel and time-based opportunity costs for patients with pain. We achieved the successful implementation of telemedicine across a pain division in an urban academic medical center with high patient satisfaction and patient cost savings.

## Introduction

### Background

Chronic pain is the most common reason for seeking medical care, with a prevalence of 50% and 10% of localized and generalized chronic pain, respectively, in the United States. Chronic pain is associated with significant occupational, functional, and psychological morbidity and accounts for an estimated annual US $61 billion in lost productivity in the United States [1]. Since the COVID-19 pandemic, chronic pain patients have experienced difficulties accessing pain care because of closures of pain clinics and limited access to in-person therapies needed for effective chronic pain management, such as psychological, medical, or physical interventions. Because of social distancing measures from the pandemic leading to inactivity and social isolation, many patients experienced additional exacerbation of their symptoms.

However, pandemic policies discouraging direct in-person contact forced pain practices to use alternative methods to deliver care to chronic and interventional patients, which led to the rapid adoption of telemedicine. To encourage telemedicine use, governments eased regulations, and Medicare [2] and other insurers temporarily established reimbursement parity with in-person visits, which commercial insurers emulated to help drive telehealth adoption and help health care providers maintain care continuity and avoid missed care [3,4]. In addition, the increasing availability of internet access and computer devices allows for greater viability of telemedicine services; 85% of households in the United States have an internet subscription and 92% have a device with computer capabilities, including smartphones [5]. Before the pandemic, telehealth was rarely used for pain management and was generally confined to pain care for military patients in remote settings [6] or trialed for a small number of patients [7-9].

### Rationale

Clinical consultations using telemedicine have been associated with patient cost savings, without a difference in clinical outcomes compared to in-person visits [10], and with health system benefits [10]. In addition, clinical consultations using telemedicine have been associated with high levels of patient satisfaction and acceptance of telehealth services, both for patients with chronic disease states [11] and those requiring postprocedural follow-up [12]. Despite the progressive adoption of telemedicine to deliver pain care, there is limited literature that has helped in the understanding of patient satisfaction with telemedicine use for pain management and its associated patient-centered time and financial savings compared to in-person visits. While the future of clinical pain practice continues to evolve to include hybrid care delivery using both in-person and telemedicine visits, there is limited telemedicine literature to help inform wider adoption of telemedicine and its associated best practices for pain management.

### Specific Objectives

Prior to the pandemic, we initiated telemedicine-enabled pain clinics for interventional patients with chronic pain at our academic medical center and community practices to reduce wait times and no-show rates and to increase patient satisfaction. During the onset of the pandemic, the majority of outpatient pain visits at the University of California, Los Angeles (UCLA) rapidly shifted to telemedicine visits. This paper focuses on the structure, implementation, and patient cost savings and satisfaction associated with our pain division’s telemedicine program. We aimed to (1) describe the UCLA Comprehensive Pain Center’s telemedicine implementation for comprehensive pain management; (2) estimate the travel time, travel distance, and time-based opportunity savings for patients using telemedicine; and (3) describe patient satisfaction using telemedicine.

## Methods

### Study Design

This was a retrospective, observational case series study of telemedicine use in a pain division at an urban academic medical center comprising the UCLA Comprehensive Pain Center and Community Pain Clinics.

### Setting

The UCLA Comprehensive Pain Center is a multidisciplinary pain management practice in Santa Monica, California. The practice is staffed by four attending physicians, a physician assistant, and a licensed clinical social worker. In addition to performing consults and follow-up visits, providers can perform procedures without image guidance, including trigger point injections, joint injections, and nerve blocks. The UCLA Comprehensive Pain Center has 10 examination rooms equipped with an examination bed and a computer with a camera for electronic medical record (EMR) access via Epic (Epic Systems Corporation) and telemedicine use. The majority of procedures are done on an outpatient basis at the Santa Monica UCLA Outpatient Surgery Center. Typical outpatient procedures include epidurals, nerve blocks, radiofrequency ablations (ie, neurolysis), joint injections, kyphoplasties and vertebroplasties, and placement of spinal cord stimulators and intrathecal pain pumps.

The UCLA Community Pain Clinics are community extensions of the UCLA Comprehensive Pain Center. There are currently nine attending physicians operating out of six office locations in the Greater Los Angeles Metropolitan Area. The community sites provide the same quality of care at a variety of geographical locations distributed throughout the Los Angeles Metropolitan Area. Outpatient procedures are performed at nearby surgery centers within the community. Patient demographics, diagnoses, procedural interventions, and telemedicine use closely resemble those seen at the Santa Monica UCLA Comprehensive Pain Center.

For both the Santa Monica UCLA Comprehensive Pain Center and the UCLA Community Pain Clinics, patients are generally referred from their primary care or surgical providers. All pain management providers perform a thorough evaluation, including a history and physical, and review all the labs, imaging, and other pertinent data. Management may involve additional diagnostic studies, medication, multidisciplinary therapy, and interventional procedures. Patients already on opioid medication constitute a significant portion of the referrals. Given the opioid epidemic and US Centers for Disease Control and Prevention guidelines for opioid management, many prescribing physicians emphasize weaning opioid usage, especially for patients on more than 60 morphine milligram equivalents. If the pain management provider agrees to manage the patient’s opioid regimen, regular follow-up is usually required every 4 weeks.

### Data Sources

We extracted demographic data from the EMR for all video visit encounters within the anesthesiology pain division from August 1, 2019, to June 30, 2020. Telemedicine visits for patients residing in California were included for analysis. During the COVID-19 pandemic in California, a “Safer at Home” directive was ordered on March 18, 2020. Before the COVID-19 era, patients used telemedicine prior to the “Safer at Home” directive; during the COVID-19 era, patients had their first telemedicine visit for pain management following the directive.

To measure patient satisfaction, we used data from a patient satisfaction survey sent to patients after their video visit. UCLA Connected Health emails each patient a patient satisfaction survey after completion of their video visit. The survey has 11 questions with responses on a 5-point Likert scale and a section for comments. From March 24 to April 22, 2020, UCLA Connected Health transitioned its survey platform from REDCap (Research Electronic Data Capture) to Qualtrics; during this period, patient satisfaction surveys were not distributed, and no patient satisfaction survey data are available for this period. All survey responses from August 1, 2019, to March 23, 2020, and from April 23 to June 30, 2020, were included for survey analysis.

### Intervention

The UCLA Comprehensive Pain Center implemented telemedicine for use in clinical care in July 2019, and we report on the period from August 1, 2019, to June 30, 2020. The UCLA Pain Division uses telemedicine for initial consultations, medication management, and postprocedural follow-up visits. In Textbox 1, we list our institution’s telemedicine eligibility criteria.

**Clinical use cases of patients with pain who are offered a telemedicine video visit.**

Clinical use cases for telemedicine:

- New patients for postoperative pain medication management
- New patients that have been referred by a spine surgeon for a specific injection
- Posthospitalization follow-up of patients
- Patients on medication management who need a medication refill or adjustment in medication
- Patients who need to discuss imaging results and next steps in their management
- Patients with recurrent pain who may need a repeat injection
- Postprocedural (ie, pain injection) follow-up of patients

Our group’s clinical experience thus far with telemedicine is that it offers a convenient care delivery option to the patient and that, generally, adequate clinical assessments can be made during a telemedicine visit. In all telemedicine use cases, the need for an in-person physical exam is made on a case-by-case basis. An abbreviated physical examination is documented in the EMR. If the source or nature of pain is unclear during the telemedicine visit, the clinician may request for the patient to come for an in-person evaluation. Other reasons the clinician may request an in-person evaluation include the following: if physical examination is required by the patient’s insurance company for procedural authorization, though many, including Medicare, do not require authorization for procedures; if there are concerning lesions on a patient’s magnetic resonance imaging scan necessitating a more comprehensive physical examination; and if urine drug screening is requested, for instance, if a substance misuse disorder is suspected. Overall, in our experience, about 10% of telemedicine visits are converted to in-person visits.

For initial telemedicine consultations, the physician elicits a history from the patient, and an abbreviated physical exam is performed remotely by asking the patient to do certain maneuvers and eliciting feedback on pain in response to these movements. Further management is decided based on the history and the abbreviated physical examination. Should a procedure be recommended, a complete physical examination is performed in the preoperative area on the day of the procedure.

The UCLA Anesthesiology Pain Division also offers the option for telemedicine visits for medication management as an alternative to in-person visits. During a telemedicine visit for medication management, the patient provides the physician with an update regarding pain levels and functionality while on the current medication regimen and reports any adverse side effects associated with the medication regimen. The physician may review pertinent imaging and diagnostic studies or order additional studies and referrals. The physician also assesses for any aberrant drug-related behaviors and checks CURES (Controlled Substance Utilization Review and Evaluation System) reports before prescribing scheduled medications. Prescriptions are primarily e-scripted to the patient’s preferred pharmacy, and patients are generally prescribed up to a 30-day supply of medication, with additional refills as appropriate.

Our institution has also used telemedicine for postprocedural follow-up. Depending on the procedure, patients are offered the option to follow up with their pain physician using telemedicine as early as 2 to 3 days to 3 to 4 weeks after the procedure. For patients who seek radiofrequency ablation, most insurance payors require two diagnostic medial branch nerve blocks to be completed with significant improvement (ie, >80% improvement in functionality and pain scale) before proceeding with radiofrequency ablation. This requires a follow-up evaluation between these staged procedures, for which we offer a telemedicine follow-up visit. For most other nonstaged procedures, procedure follow-ups can also be completed via telemedicine. During a postprocedural follow-up telemedicine visit, the clinician will inquire about any improvement after the procedure and any adverse side effects associated with the procedure. The patient will be asked to comment on improvement in functionality, if any, as a result of the procedure. The patient and the physician would discuss the next steps in clinical management, including medications, the potential for repeat procedures, additional procedures, and referrals to other specialists.

**Figure 1:**
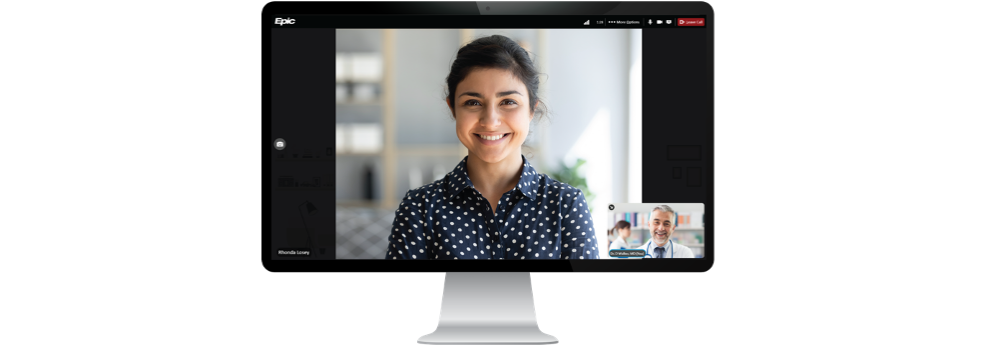
Representative illustration of the interface used for pain telemedicine visits at the UCLA Comprehensive Pain Center (© 2021 Epic Systems Corporation). UCLA: University of California, Los Angeles.

UCLA Health uses Vidyo videoconferencing software (Vidyo, Inc) for its telemedicine video visits. Telemedicine visits are scheduled within Epic, and providers log in to the video visit from the Epic clinic schedule. UCLA Health uses a mobile-only option for patients who use the Epic MyChart app (Epic Systems Corporation) on their smartphone or tablet to log in to the myUCLAhealth patient portal. After logging in to the Epic MyChart app, patients are prompted with a “begin visit” button at the top of the opening webpage. Patients wait in a virtual “waiting room” until the provider logs on to the video interface. Figure 1 illustrates the video interface for the telemedicine visits.

### Outcomes and Definitions

We evaluated patient cost savings and satisfaction associated with telemedicine use in our patient population. To calculate patient costs saved by using telemedicine for care as opposed to an in-person visit, the patient’s total cost for an in-person visit was estimated by calculating and totaling the total fuel cost for travel by car to a clinic visit, parking cost, and time-based opportunity cost, based on previously published methods [12,13]. The round-trip travel time and round-trip travel distance were calculated using each patient’s originating zip code to that of their pain management clinic using Google Maps’ Distance Matrix application programming interface (Google). Travel time with traffic was estimated by assuming afternoon telemedicine encounters beginning at 2 PM. Given that driving is much more common than the use of public transportation in the Greater Los Angeles Area [14], driving time was used to estimate travel time. To calculate the total travel fuel cost, we used the median value for regular fuel cost in California during the time period included in this study (US $3.42/gal) [15] and fuel economy (24.9 miles/gal) [16] among California vehicles. Day parking costs at the UCLA Medical Center Westwood, Santa Monica, and the community practices were US $13, US $20, and US $0, respectively, at the time of the study. To estimate the time-based opportunity cost for travel to an in-person visit, we calculated the patient’s estimated earnings per hour based on their zip code, and then multiplied their estimated earnings per hour by their estimated round-trip travel time. To estimate patient earnings per hour by zip code, we used the Internal Revenue Service’s Statistics of Income (IRS SOI) program’s Individual Income Tax Statistics–2017 ZIP Code Data [17], which was the most recent data set publicly available. We adjusted for inflation from 2017 to 2020 US dollars using January data from the US Bureau of Labor Statistics’ (BLS) Consumer Price Index [18].

To estimate patient earnings per hour by zip code based on the IRS SOI, total annual earnings within a zip code were first calculated as the sum of two income categories: (1) business or professional income and (2) salaries and wages. We defined earnings to include wages and income from running a business, a more comprehensive definition of labor earnings. In contrast, wages listed in employer databases do not reflect patients’ income from running a business. For individual returns, annual earnings per person can be calculated by dividing total annual earnings by the number of individual returns within the zip code. For joint filings, earnings per person must be calculated by dividing total earnings by twice the number of filings, since each filing reflects two people’s combined earnings. Within each zip code, annual earnings per person are calculated using a denominator that reflects the share of filings that were individual versus joint returns [19,20]. In particular, we used the following calculation to estimate annual earnings per person within each zip code:

Annual earnings = the sum of average annual earnings / ([(2 × % married filing jointly) + % individual returns] × the total number of returns)

Finally, to estimate earnings per hour, we divided the estimated annual earnings per person by 2000 hours per year. We assumed 2000 working hours per year, which equals 40 hours per week times 50 weeks per year. This is similar to the annual work hours assumption used by the BLS when it estimates hourly wages from surveys of employers: the BLS uses an estimate of 2080 hours per year [21]. Notably, our estimate allows for a larger fraction of the patient population to be unemployed, out of the workforce, or working part time. To the extent that patients both earn less annually and work fewer hours per year than average, these factors may offset each other with respect to hourly earnings calculations.

To estimate the time-based opportunity savings, we multiplied the round-trip travel time by the patient’s estimated hourly earnings by zip code; for this pain population, the median hourly earnings were US $28 per hour. Sensitivity analyses were conducted by varying the fuel cost (US $3.20-$3.80/gal), the median round-trip distance (5-200 miles), and the fuel economy (15-60 miles/gal) to characterize the range of travel costs, round-trip travel distance, and hourly earnings. A subset of our patients had serial visits for chronic pain management, and we conducted a sensitivity analysis varying hourly earnings and number of visits to understand savings with telemedicine over chronic care management.

Patient characteristics and study variables were summarized using mean (SD), median (IQR), or frequency (%), unless otherwise noted, using the Python programming language (Python Software Foundation [22]). In order to summarize the distribution of each of our patient characteristic variables or outcome variables, we first assessed the distributions visually. For variables that were approximately normally distributed, we used mean and SD as our summary statistics. For variables that had a skewed distribution, we elected to use median and IQR. Statistical comparisons between groups (ie, patients from the pre–COVID-19 era and those from the COVID-19 era) were assessed using the independent-samples *t* test for continuous variables (2-tailed) and the chi-square test for categorical variables (eg, gender and race). *P* values less than .05 were considered statistically significant.

### Ethical Considerations

Institutional Review Board (IRB) approval was obtained but given exempt status for the purposes of analyzing and retrospectively reporting our results for quality improvement (IRB #20-000573).

## Results

### Demographics

We completed 2948 telehealth video visits with 1398 patients. The mean age of the total patient sample was 56 (SD 16) years. Patients from the pre–COVID-19 era were, on average, younger than patients from the COVID-19 era (52 [SD 14] years vs 56 [SD 16] years, respectively; *P*<.001). There was no significant difference in the distribution of race between cohorts from the pre–COVID-19 era and the COVID-19 era. Additional patient demographic characteristics are presented in Table 1.

**Table 1:** Demographic characteristics of patients who completed a video visit within the anesthesiology pain division between August 2019 and June 2020.

Characteristic		All patients from completed encounters (N=1398)	Patients from pre–COVID-19 era (n=219)	Patients from COVID-19 era (n=1179)	*P* value^a^
Video visits, n (%)^b^		2948 (100)	511 (17.3)	2120 (71.9)	N/A^c^
**Age (years**)					
	Mean (SD)	56 (16)	52 (14)	56 (16)	<.001
	Median (IQR)	57 (44-67)	53 (43-62)	58 (45-68)	N/A
**Gender, n (%)**					
	Male	545 (39.0)	84 (38.4)	461 (39.0)	.84
	Female	853 (61.0)	135 (61.6)	718 (60.9)	
Round-trip travel time (minutes), median (IQR)		69 (46-101)	81 (52-113)	68 (44-99)	.002
Round-trip travel distance (miles), median (IQR)		26 (13-55)	28 (13-54)	26 (13-57)	.07
Earnings (US $/hour), median (IQR)		28 (21-39)	31 (22-44)	28 (21-37)	.03
Total savings per video visit (US $), median (IQR)		52 (36-75)	66 (49-108)	51 (31-74)	<.001
**Self-reported race, n (%)**					
	White or Caucasian	1012 (72.4)	169 (77.2)	843 (72.0)	.54
	Other race	142 (10.2)	18 (8.2)	124 (10.5)	
	Black	107 (7.7)	13 (5.9)	94 (8.0)	
	Declined to specify	51 (3.6)	7 (3.2)	44 (3.7)	
	Asian	50 (3.6)	9 (4.1)	41 (3.5)	
	Unknown	22 (1.6)	2 (0.9)	20 (1.7)	
	American Indian or Alaska Native	10 (0.7)	0 (0)	10 (0.8)	
	Native Hawaiian or other Pacific Islander	4 (0.3)	1 (0.5)	3 (0.3)	

^a^*P* values were based on statistical comparisons between groups, which were assessed using the independent-samples *t* test for continuous variables (2-tailed) and the chi-square test for categorical variables. *P* values for categorical variables are reported in the top row of that group.

^b^Percentages in this row only are based on the total number of video visits (N=2948).

^c^N/A: not applicable; statistical comparisons were not performed for “video visits” or median “age.”

### Telemedicine No-show Data

A total of 3006 telehealth video visits were scheduled for 1419 patients. Of 3006 scheduled telemedicine visits, there were 58 (1.9%) no-shows and 2948 (98.1%) successfully completed telehealth visits.

### Patient Satisfaction Data

**Figure 2:**
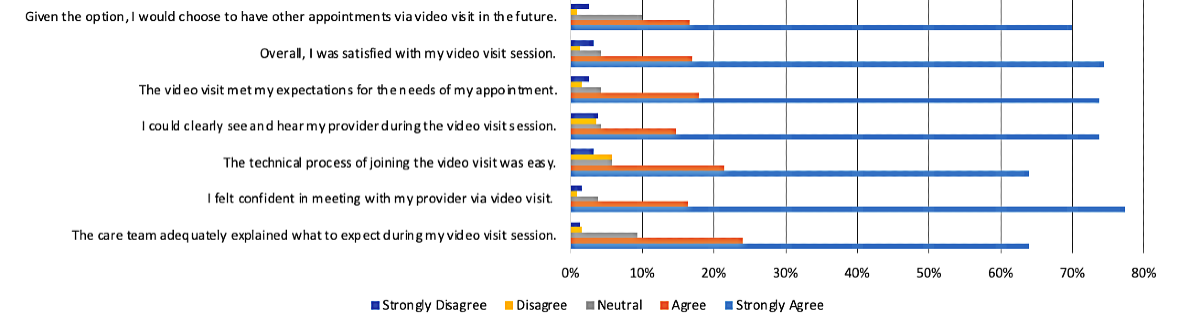
UCLA Health telemedicine patient satisfaction survey results (n=313 surveys). UCLA: University of California, Los Angeles.

Of the 2192 video visit encounters for which a patient experience survey was emailed, there were 313 completed patient experience surveys (response rate of 14.3%). Patient satisfaction for using telehealth for pain management was high. Out of 313 survey responders, 286 (91.4%) either “agreed” or “strongly agreed” that they were satisfied with a video visit for care management, and 293 (93.6%) survey responders either “agreed” or “strongly agreed” that they felt confident in meeting with their provider via a video visit. Out of 313 survey responders, 271 (86.6%) said that they either “agreed” or “strongly agreed” that they would prefer future video visits for pain management care. We present the results of the patient experience survey in Figure 2.

### Travel Distance and Time Saved

**Figure 3:**
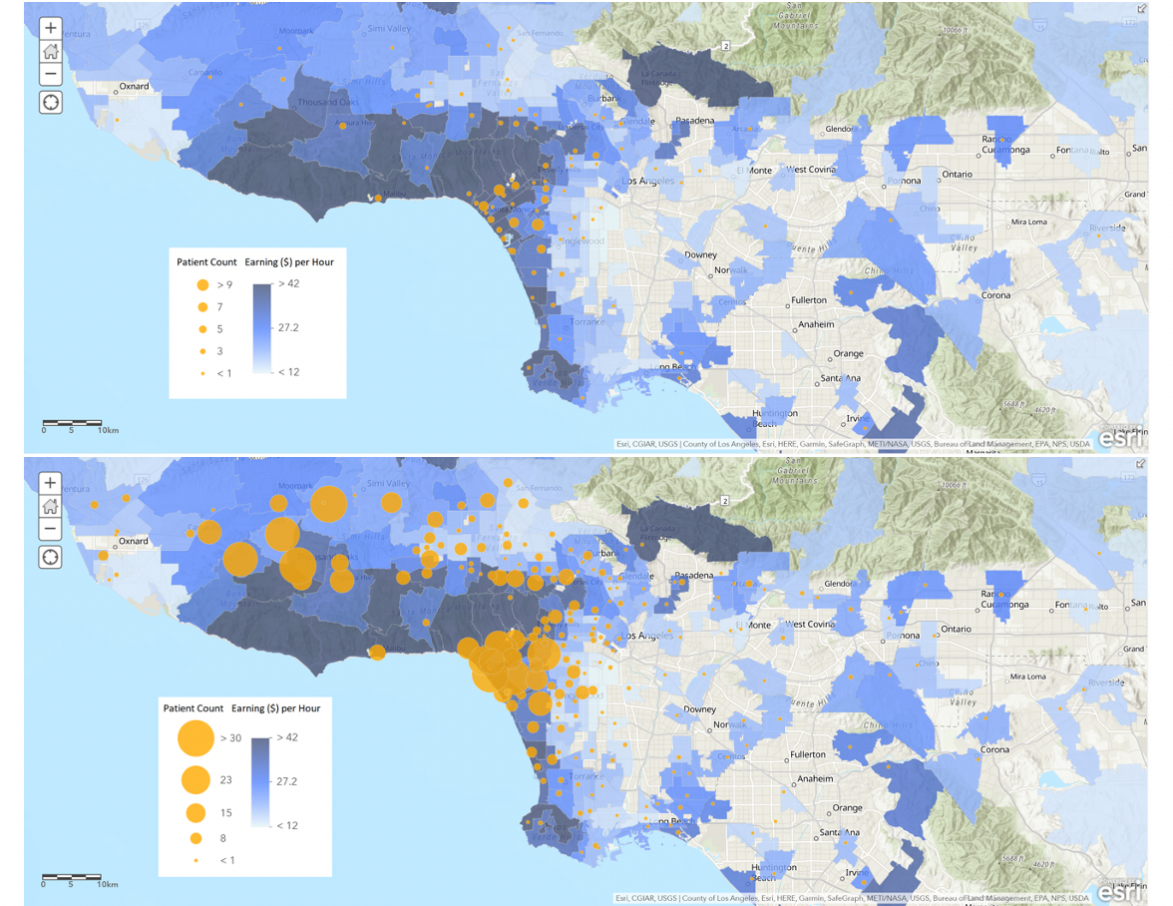
Geographical distribution of patients from the pre–COVID-19 era (top image) and patients from the COVID-19 era (bottom image).

As calculated from the patients’ home zip codes to their pain providers’ clinics, the median round-trip travel distance was 26 miles (IQR 13-51). The median round-trip travel time in afternoon traffic conditions was 69 minutes (IQR 46-101). Patients from the pre–COVID-19 era experienced longer travel times than patients from the COVID-19 era (*P=*.002). Figure 3 presents the geographical distribution of patients who participated in a telemedicine video visit.

### Patient Cost Savings With Telemedicine

We estimated direct and time-based opportunity savings that patients obtained from a telemedicine video visit. Patients experienced direct savings in fuel and parking costs. Our results suggest that patients experienced a median direct savings of US $22. Because differences in regional fuel cost, fuel economy, and distance traveled affect the total savings, we conducted sensitivity analyses by varying round-trip driving distance, fuel economy, and fuel prices per gallon (Figures S1 and S2 in Multimedia Appendix 1).

Patients also experienced time-based opportunity savings. Based on this cohort’s zip code residence, their median hourly earnings were US $28 (IQR US $21-$39) per hour, with subsequent median time-based opportunity savings of US $32, bringing the estimated median total savings per telemedicine video visit to US $52 (IQR US $36-$75). Patients from the pre–COVID-19 era had higher hourly earnings (*P=.*03) and experienced greater estimated total savings than patients from the COVID-19 era (*P<*.001). Sensitivity analyses were conducted to reflect the value of a telemedicine encounter based on hourly earnings (Figure S3 in Multimedia Appendix 1). Patients who received serial telemedicine care had a median number of 3 visits and saved a median of US $156. We conducted a sensitivity analysis evaluating total savings for multiple telemedicine visits for chronic pain management care (Figure S4 in Multimedia Appendix 1).

## Discussion

### Principal Findings

This study examined telemedicine encounters within the anesthesiology pain division of an urban academic health care system and its affiliate community practices, both before and after implementing the COVID-19 “Safer at Home” order, focusing on patient savings and satisfaction. Patients who attended telemedicine visits avoided an estimated median round-trip driving distance of 26 miles and a median travel time of 69 minutes during afternoon traffic conditions. Within the sample, the median hourly earnings were US $28 per hour. Patients saved a median of US $22 on gas and parking and a total of US $52 per telemedicine visit based on estimated hourly earnings and travel time. Patients who were evaluated serially with telemedicine for medication management saved a median of US $156 over a median of 3 visits. Out of 313 patients surveyed, 91.4% (n=286) were satisfied with their telemedicine experience.

### Study Strengths

We introduced our telemedicine program for all the patients seen in our health care system meeting our eligibility criteria, and not for a narrow subset of patients, improving the generalizability of our findings to a general pain management population. Inclusion in our study of patients seen in both academic and community-based practices also improved our findings’ generalizability. Our study included a relatively high number of patients, making the findings more robust. Our use of IRS SOI data to estimate hourly earnings by zip code, rather than using citywide median incomes, likely strengthened the accuracy of our cost savings estimate.

### Comparison With Prior Studies

Other studies have established the significant increase in telemedicine adoption since the beginning of the COVID-19 pandemic [23,24] as well as the high level of patient satisfaction with telemedicine visits [23]. Several studies have specifically examined the implementation of a telemedicine program for patients with chronic pain [7-9,25], some of which reported a high degree of patient satisfaction [7,8] and significant patient cost savings [8,9] as we found in our study, though measured in sample sizes totaling less than 50 patients. Pronovost et al [9] found a total patient cost savings per patient of US $310 more than we found in our study, though this was the cumulative savings that patients experienced longitudinally over months rather than with a single visit as we calculated; additionally, only patients with a travel distance greater than 100 kilometers were included, likely inflating travel costs for in-person visits. The telemedicine program introduced in Hanna et al [7] was only offered to a subset of patients living on an island, with the study’s pain center only accessible by sea or air, possibly making patients more satisfied with telemedicine due to the lack of ease of access to in-person health care. Similarly, Peng et al [8] also reported satisfaction with telemedicine, but in a sample of patients with an average home-to-clinic travel distance of 314 kilometers, making the ease of telemedicine more pronounced in this population. Song et al [25] described telemedicine use for pain management during the COVID-19 pandemic as we did, but did not include any quantified metrics examining the benefit of telemedicine in this context. To our knowledge, ours was the only study that investigated differences in patient characteristics between those using telemedicine for chronic pain before and after the beginning of the COVID-19 pandemic.

### Study Limitations

Our methods only took into account the use of cars as a transportation mode. We did not look at other means of transportation (eg, bus, ride-sharing, and flights) when calculating travel costs. Furthermore, ideally, our time-based opportunity cost analysis would quantify patient savings at the individual level. While our approach provided a more refined way of calculating time-based opportunity cost, our approach was limited in that even within a zip code, earnings varied for particular patients, and our method did not, therefore, perfectly capture patient earnings. Our time-based opportunity cost analysis also did not consider patients who may be unemployed, unable to work, or retired, which may represent hourly earnings of $0 per hour and for whom total savings may only reflect travel costs. We also compared the observational cohorts on a few characteristics that we could abstract from the EMR, leaving the potential for unmeasured confounding factors.

### Study Implications

Patient savings are an essential component of the financial benefits of telemedicine. These savings should ideally be quantified at an individual patient level to reflect heterogeneous populations of patients with different opportunity costs associated with travel for in-person visits. Opportunity costs are typically estimated based on hourly wage rates or hourly earnings, but such individual income data are not collected by health systems. In this study, we estimated the earnings of our sample of patients with pain using a novel approach that used publicly available IRS SOI data, which allowed us to estimate hourly earnings by zip code. Earnings estimated based on patient zip codes are more accurate than using citywide or national median incomes or wages, which is the measure used in previous studies [12,26,27]. This is because earnings vary by geography, and people with similar incomes tend to cluster together geographically in particular zip codes. Medical centers in specific locations will often serve specific patient populations with higher or lower earnings than the citywide median wage or national averages; thus, a national or citywide median wage will tend to underestimate the earnings of a patient population living primarily in high-income areas and overestimate the earnings of a patient population living primarily in low-income areas. Our approach allows telemedicine’s financial benefits to vary for different medical centers and different practice areas that serve populations with different income levels, facilitating a more accurate estimate of patient savings.

Our data suggest that patients who stood to benefit the most from adopting telemedicine—because they have higher hourly earnings and, therefore, higher time-based opportunity costs of in-person visits—were early adopters of telemedicine. Patients from the pre–COVID-19 era had higher total savings compared to the patients from the COVID-19 era (*P<.*001). Telemedicine offers value for patients in the form of time-based opportunity savings, especially for patients who must travel long distances, experience traffic, or have high time-based opportunity costs from missed work. A subset of patients had multiple video visits for medication management. Figure S4 in Multimedia Appendix 1 is a sensitivity analysis showing savings over serial telemedicine care, suggesting that telemedicine may provide a cost-effective means for obtaining continuity of pain management care.

Patient satisfaction as a measure of quality of care is also a key part of value-based care. It has been associated with the success of telemedicine initiatives [28], patient retention [29], and treatment plan adherence [30]. Our study revealed high patient satisfaction with telemedicine, with 86.6% of patients (271/313) responding that they would elect to use telemedicine again. As the pandemic abates, pain providers could consider developing a hybrid model of care using telemedicine and in-person visits.

### Telehealth Sustainability for Pain Management and Future Challenges

Consensus recommendations from panels of adult chronic pain health professionals have identified the value of telemedicine to manage pain [31,32], but sustained telemedicine use by pain practices will be dependent on provider acceptance, a supportive reimbursement policy environment, and patient technical literacy. Pain clinicians will need to determine the right criteria for telemedicine and in-person care moving forward, and clinical practice guidelines and malpractice policies will need to be updated to incorporate telemedicine and remote monitoring [31]. The financial implications of telehealth for pain practices’ overhead costs, including different staffing models and the need for office leases, will need to be understood in order to find areas for possible cost savings and revenue for practices [33]. Should telehealth continue to be a valued care delivery option for pain management, policy makers will need to expand reimbursement to promote high-volume telemedicine use, and quantifying patient cost savings could assist in policy development for reimbursement of telemedicine services [34]. Expanded reimbursement may include creating billing codes or payment models that take into account the additional work required to offer telemedicine visits, such as technical triage or having staff prepare patients for a telemedicine visit.

Finally, challenges with using telemedicine at the patient level have begun to come to light since the COVID-19 pandemic. Access to telemedicine involves different economic and social factors, and chronic and interventional patients will need access to telecommunications technologies [35] as well as the ability to use them effectively. Pain practices will need to understand their patients’ technological literacy, their patients’ limitations around access to the internet [36] and devices, and the characteristics of their patients who do not use telemedicine [37,38].

### Conclusions

In this study, we aimed to describe the implementation and evaluation of our adult anesthesia pain division’s standardized telemedicine practice guidelines for adult chronic and interventional patients with pain and to estimate total patient cost savings and satisfaction with telemedicine. We found that per visit, patients saved US $52, on average, taking into account both actual cost and time-based opportunity cost and that a high proportion of patients surveyed were happy with their telemedicine experience. Chronic pain causes significant suffering and a reduced quality of life, especially in the setting of a pandemic, but telemedicine provides efficient and cost-effective care to patients with chronic pain. Our telemedicine initiative was built on our academic hospital’s comprehensive pain center and the community clinics’ capacity to treat and care for patients with pain needs; our findings suggest that expanding the use of telemedicine for pain management may save patients time, reduce costs, and provide high patient satisfaction. Following the COVID-19 pandemic, we anticipate that telemedicine management for patients with pain will continue to evolve as health care systems strive to improve population health and improve care access for patients with pain needs.

## Appendix

Multimedia Appendix 1 Sensitivity analyses.

## Abbreviations

BLS: Bureau of Labor Statistics
CURES: Controlled Substance Utilization Review and Evaluation System
EMR: electronic medical record
IRB: Institutional Review Board
IRS SOI: Internal Revenue Service’s Statistics of Income
REDCap: Research Electronic Data Capture
UCLA: University of California, Los Angeles
