# Supplementary material for: Evaluation of Telemedicine Use for Anesthesiology Pain Division: Retrospective, Observational Case Series Study
Source: JMIR Perioper Med. 2022 Apr 27;5(1):e33926. doi: 10.2196/33926 (PMC9049645; doi:10.2196/33926)
Supplement: Multimedia Appendix 1 [file periop_v5i1e33926_app1.docx]

**Multimedia Appendix 1.** Sensitivity analyses.

**Figure S1:** [Sensitivity analysis of total gas and parking savings incurred to the patient, assuming a median fuel economy of 24.9 miles/gallon and US $20 parking.](https://periop.jmir.org/api/download?filename=947e60e18d2721f81f7c10c168431ace.pdf&alt_name=33926-577320-1-SP.pdf)

**Figure S2:** [Sensitivity analysis of total gas and parking savings, assuming a median round-trip distance of 26 miles and US $20 parking.](https://periop.jmir.org/api/download?filename=d8541568b2885b647943167c9980e9bd.pdf&alt_name=33926-577321-1-SP.pdf)

**Figure S3:** [Sensitivity analysis of patient time-based opportunity savings, assuming median hourly earnings of US $28/hour and median round-trip driving time of 69 minutes.](https://periop.jmir.org/api/download?filename=34ed2ae5879f46dcac88bfe38e8c6935.pdf&alt_name=33926-577323-1-SP.pdf)

**Figure S4:** [Sensitivity analysis of total savings for chronic pain management assuming fuel costs of US $3.42/gallon, fuel economy of 24.9 miles/gallon, median hourly earnings of US $28/hour, and median round-trip distance of 26 miles.](https://periop.jmir.org/api/download?filename=f566372bfae852f3f958c017e7dced00.pdf&alt_name=33926-577324-1-SP.pdf)
